# Decisional balance and processes of change in community-recruited with moderate-high versus mild severity of cannabis dependence

**DOI:** 10.1371/journal.pone.0188476

**Published:** 2017-12-04

**Authors:** Francisca López-Torrecillas, Eva María López-Quirantes, Antonio Maldonado, Natalia Albein-Urios, Mª del Mar Rueda, Antonio Verdejo-Garcia

**Affiliations:** 1 Learning, Emotion and Decision Group, Department of Experimental Psychology, University of Granada, Granada, Spain; 2 Center Research Mind Brain and Behavior (CIMCYC), University of Granada, Grenada, Spain; 3 School of Psychology, Deakin University, Burwood, Victoria, Australia; 4 Department of Statistics and Operations Research, University of Granada, Granada, Spain; 5 School of Psychological Sciences, Monash University, Melbourne, Australia; Waseda University, JAPAN

## Abstract

Decisional Balance and Processes of Change are generally addressed in motivational interventions for the treatment of cannabis use disorders. However, specific aspects of these multifaceted constructs, with greater relevance for severe cannabis users, need to be ascertained to enable better interventions. This study aimed to compare the different facets of decisional balance and processes of change between mild and severe cannabis users in a community-based sample of young undergraduates. Thirty-one severe cannabis users and 31 mild cannabis users, indicated with the Severity of Dependence Scale, were assessed using the Decisional Balance Questionnaire (DBQ) and the Processes of Change Questionnaire (PCQ). We found that severe cannabis users had higher scores in the DBQ dimensions of Utilitarian Gains for the Self, Utilitarian Gains for Significant Others, and Self-approval, as well as in the total subscale of Gains but not Losses. The group of severe cannabis users also had higher scores in the PCQ dimensions of Self-revaluations and Counter-conditioning. Our results pinpoint specific dimensions of Decisional Balance and Processes of Change that are endorsed by severe cannabis users. This knowledge could be applied to inform motivational interventions targeting severe cannabis users.

## Introduction

Cannabis is the most commonly used illegal drug in Europe; 14.6% of young adults (ages 15 to 34) have used cannabis within the past month (European Monitoring Centre for Drugs and Drug Addiction [1]. Cannabis use has been associated with different behavioral, cognitive and emotional deficits, including apathy, disinhibition and executive dysfunction [2, 3]. In addition, cannabis use is associated with significant negative social consequences, may act as a gateway to the use of other substances, and can trigger or worsen psychotic symptoms [4, 5]. Recent calls for research have highlighted that severe cannabis users are more likely to experience these symptoms, and hence more likely to request treatment for cannabis use disorders [6–8].

Psychological treatments for cannabis use disorders have been largely grounded on the Transtheoretical Model of Behavior Change. This framework fostered the application of motivational interventions, which capitalise on motivational factors that can stimulate cannabis users to quit the drug [9–11]. The model emphasises the relevance of motivational factors, such as Decisional Balance and Processes of Change, as the leverage to facilitate behavioural change [12–14].

Decisional Balance involves weighing up the perceived advantages (gains) and disadvantages (losses) of using cannabis versus quitting cannabis. Cognitive processes linked to disadvantages of smoking and gains of quitting have shown to shift the decisional balance in favour of quitting as indicated by research in nicotine dependence [15]. However, these cognitive processes are multidimensional, including both gains and losses in four different aspects: 1) Anticipated utilitarian effects for the self; 2) Anticipated utilitarian effects for significant others; 3) Self approval; 4) Anticipated effect on how one is regarded by significant others. Empirical evidence suggests that in individuals that are ready to change their behavior, the cognitive processes linked to the benefits exceed the ones of the costs [16]. Accordingly, decisional balance measures have been shown to predict clinical outcomes in nicotine use [17] and alcohol use disorders [12, 18].

The Processes of Change represent 10 different behavioural and experiential strategies for changing behaviour. They have been classified in cognitive-affective processes (defined as changes in the way people think and feel about their smoking) versus behavioural processes (defined as coping strategies that are utilized to change cannabis use behavior). Cognitive-affective processes include consciousness raising (CR), Dramatic Relief (DR), Environmental Re-evaluation (ER), Self-revaluation (SR), and Social Liberation (SO). Behavioural processes include Stimulus Control (SC), Counter Conditioning (CC), Reinforcement Management (RM), Self- Liberation (SL), and Helping Relationships (HR). Cognitive-affective processes are more often utilized during early stages of change, fostering awareness and control of thoughts, feelings and health-related goals [13, 14] and behavioural processes are more typical in later stages of change [19, 20].

Decisional Balance and Processes of Change facets are typically addressed in some of the most successful treatment interventions for cannabis dependence, such as motivational interventions. However, it would be relevant to determine which of these facets are actually endorsed by severe cannabis users, as the facets that better resonate with their own motivations will be likely to have stronger therapeutic effects. To address this unmet need, this study aims to compare Decisional Balance and Processes of Change in severe versus mild to moderate cannabis users in a community-based sample of young undergraduates. This comparison will identify the specific dimensions of Decisional Balance and Processes of Change that are endorsed by severe cannabis users, which are similar to treatment-seeking populations. Therefore, the results obtained in this community sample can be useful to inform the contents of motivational interventions in clinical samples. We hypothesised that severe cannabis users compared to mild users: 1) would endorse less gains and more losses related to using cannabis; 2) would endorse more behavioral and less cognitive processes of change.

## Methods

### Participants

The sample comprised community-dwelling young adults between 17 and 45 years of age, recruited from the student population of the University of Granada. Participants were recruited by university faculty during class breaks and were selected using a probabilistic sampling design. In particular, a cluster stratified sample design was adopted. Strata were based on the different university faculties. Cluster samples were extracted such that majors and years of study were represented in proportion to the total number of students in each faculty. Finally all students of the cluster sample were included in the final sample. There were 856 student participants recruited between September 2013 and June 2014. They were informed about the aims of the study and provided signed informed consent. Ethical approval was obtained from the Research Ethics Committee from the University of Granada.

All 856 participants completed the Severity of Dependence Scale (SDS) and 115 reported that they were cannabis users. We divided these 115 cannabis users in two groups. The first group comprised cannabis users of moderate severity (n = 84) and the second group comprised severe cannabis users (n = 31) as indicated by the SDS cut-off defined by Cuenca-Royo et al. [21]. In order to get similar sample sizes for group comparisons, we selected a random subsample of 31 moderate cannabis users by simple random sampling without replacement. The socio-demographic characteristics and variables related to cannabis smoking in the final sample can be seen in Table 1. We found sex differences in the severity of cannabis use: the moderate group consisted mostly of women and the severe group consisted mostly of men.

**Table 1:** Baseline demographic and variables related to cannabis smoking of the participants classified in each of these categories (moderate versus severe).

Variables	MODERATE	SEVERE	*F/ χ* ^*2*^
**Age (mean and SD)**	20.81 (2.09)	22.58 (3.23)	6.588*Ns*
**Gender (N)**			
*Male (Female)*	10 (21)	24 (7)	12.765**
Tobacco dependence [Fagerström] (mean and SD)	.87 (1.56)	2.10 (2.25)	6.178*Ns*

**p< 0.001

### Measures

The protocol used is included in Supporting information (S1 Table) and includes the following measures.

#### The Severity of Dependence Scale (SDS [22])

The severity of cannabis use was measured using the Spanish version of the SDS [21]. The SDS is a 5-item questionnaire with a 4-point Likert scales (from 0 to 3) that has been reported to be a reliable and valid screening instrument for the severity of dependence in a population of cannabis users. Total scores range from 0 to 15, with 15 representing the highest level of dependency. The psychometric properties of the SDS have been well established in adult and adolescent populations. It demonstrates high test-retest correlations and good internal consistency [23]. The cut-off scores vary between 2 and 4, where 4 was adopted to define cannabis dependence with optimal discrimination [24]. The psychometric properties of the Spanish version show high internal consistency (alpha = 0.82) and test-retest reliability (ICC = 0.83). The cut-offs recommended are 3 to define moderate cannabis dependence and 7 for severe cannabis dependence [21].

#### Fagerström Test for Nicotine Dependence (FTND [25])

This instrument measures the intensity of physical addiction to nicotine. We was used the Spanish version [26]. The test is composed of 6 items with two or four response alternatives that evaluates the quantity of cigarettes consumed, the compulsion and dependence. The total score ranges between 0 and 10, where 1–2 = very low dependence; 3–4 = low dependence; 5 = medium dependence; 6–7 = high dependence; 8–10 = very high dependence.

#### Decisional balance for cannabis abusers [16]

In the context of cannabis use the Decisional Balance questionnaire evaluates the costs and benefits (also referred to as pros and cons) of continuing to use cannabis [27, 28]. It is a 42-item Likert scale questionnaire assessing advantages (21 items) and disadvantages (21 items) associated with cannabis dependence. Participants respond using a 5-point Likert scale indicating the importance of various statements for the person’s decision to smoke cannabis (1 = not important at all, 2 = slightly important, 3 = moderately important, 4 = very important, 5 = extremely important). The Decisional Balance questionnaire can identify patients who are motivated or unmotivated to quit or undergo treatment [16, 29].

#### The Processes of Change Questionnaire (PCQ [30])

This questionnaire evaluates the Process of Change as a personal mechanism that permits progression from one stage to another. It includes 30 items that evaluates 10 different processes using a 5-point Likert scale that measures the frequency of use in the past month (1 = Never to 5 = Repeatedly). This questionnaire was designed to assess the frequency in which participants make use of experiential processes (consciousness raising, self-reevaluation, dramatic relief, environmental reevaluation, and social liberation), and behavioral processes (counter-conditioning, stimulus control, reinforcement management, self-liberation, and helping relationships) to change cannabis abuse. Internal consistency was established with alpha coefficients ranging from .69 to .92 [31–34].

### Statistical analyses

We conducted two multivariate analyses of variance (MANOVA) using Group as the independent variable (as defined by the SDS cut-offs: Severe versus Mild to moderate users) and the scores of the different dimensions of Decisional Balance (DBQ) and Processes of Change (PCQ) as the two dependent variables. These MANOVAs were followed by univariate between-group ANOVAs on the different dimensions of the DBQ and the PCQ. All analyses were replicated using sex and tobacco dependence indicated by the Fagerström Test as covariates.

## Results

We found a significant effect of Group on Decisional Balance (Wilks' Lambda = 0.051, F_11,50_ = 83.900; *p<* 0.001; *Eta* = 0.949). Univariate tests of between-group effects revealed significant effects of Group on the total score of Gains (*F*_1,60_ = 20.188; Mean Squared Error [Mce] = 2567.76; *p<* 0.001; Eta = 0.252) and in the specific dimensions of Utilitarian Gains for the Self (*F*_1,60_ = 21.841; Mce = 276.79; *p<* 0.001; Eta = 0.267), Utilitarian Gains for Significant Others (*F*_1,60_ = 9.622; Mce = 60.016; *p<* 0.003; Eta = 0.138) and Self-approval (*F*_1,60_ = 12,281; Mce = 236.145; *p<* 0.001; Eta = 0.170). In all cases, the severe group had higher scores than the mild group. There were no significant differences in Losses. Mean, standard deviations and significance are presented in Table 2.

**Table 2:** Mean and typical deviation to evaluate status cannabis abuse decisional balance and process of change.

DECISIONAL BALANCE	GROUPS CANNABIS		*F*	Eta
	MODERATE	SEVERE		
	MEAN (SD)	MEAN (SD)		
**Utilitarian gains for self**	**8.19 (3.2)**	**12.42 (4.03)**	21.841**	**.267**
**Utilitarian gains for significant others**	**5.23 (1.71)**	**7.19 (3.09)**	9.622*	**.138**
**Self-approval**	**8.71 (4.54)**	**12.61 (4.22)**	12.281*	**.170**
Gains social approval	6.35 (2.09)	7.94 (3.40)	4.872*Ns*	.075
Gains special items	2.77 (1.38)	3.77 (1.84)	5.855*Ns*	.089
**TOTAL GAINS**	**31.26 (9.78)**	**44.13 12.60)**	20.188**	**.252**
Utilitarian losses for self	11.03 (5.70)	14.10 (3.69)	6.303*Ns*	.095
Utilitarian losses for significant others	8.23 (4.62)	9.74 (3.66)	2.049*Ns*	.033
Self-disapproval	9.39 (4.54)	11.13 (3.23)	3.032*Ns*	.048
Losses social disapproval	8.23 (5.12)	10.65 (3.08)	5.075*Ns*	.078
TOTAL LOSSES	36.90 (18.60)	45.61 (10.60)	5.131*Ns*	.079
**PROCESS OF CHANGE**				
Consciousness Raising	4.13 (4.46)	5.65 (3.79)	2.078 *Ns*	.033
**Self Re-evaluation**	**1.48 (2.76)**	**4.42 (3.24)**	14.751**	**.197**
Environment Re-evaluation	2.23 (3.26)	2.23 (3.26)	2.716 *Ns*	.043
Dramatic Relief	1.35 (2.37)	2.19 (2.10	2.167 *Ns*	.035
Self- liberation	2.58 (3.90)	5.42 (3.87)	4.343 *Ns*	.067
Social Liberation	3.65 (3.99)	5.42 (3.87)	3.159 *Ns*	.050
Reinforcement management	1.81 (3.48)	3.26 (2.67)	3.398 *Ns*	.054
Helping Relationships	2.16 (3.44)	4.77 (3.60)	8.539 *Ns*	.125
**Counter-conditioning**	**2.23 (3.50)**	**4.87 (2.99)**	10.249*	**.146**
Stimulus Control	1.52 (2.71)	1.68 (2.45)	.060 *Ns*	.001

**p< 0.001

*p< 0.01

We also found a significant effect of Group on Process of Change (Wilks' Lambda = 0.357, F_10,51_ = 9.175; *p* < 0.001; *Eta* = 0.643). Univariate tests of between-group effects revealed significant effects of Group on Self-Reevaluation (*F*_1,60_ = 14.751; Mce = 133.565; *p<* 0.001; Eta = 0.197) and Counter-Conditioning (*F*_1,60_ = 10.249; Mce = 108.452; *p<* 0.01; Eta = 0. 146). In all cases, the severe group had higher scores than the mild group. Mean, standard deviations and significance are presented in Table 2.

Covariates analyses including tobacco dependence and sex did not changed the above-reported findings.

## Discussion

The aim of this study was to compare the different dimensions of Decisional Balance and Processes of Change in community-recruited participants with severe versus mild cannabis dependence, as indicated by the SDS. With regard to Decisional Balance we found that the severe group had higher scores in Utilitarian Gains for the Self, Utilitarian Gains for Significant Others, Self-approval and total Gains. Both severe and mild users perceived similar losses. With regard to processes of change we found that the severe group had higher scores in Self-revaluation and Counter-conditioning. These findings suggest that severe cannabis users are more aware of the potential gains of quitting the drug, and make more use of behavioral processes of change, compared to mild users. They also identify the specific processes that they use to foster self-change, which may be relevant to inform the contents of motivational interventions.

The Decisional Balance findings suggest that severe cannabis users can better perceive the importance of specific gains associated with cannabis use cessation. Utilitarian Gains for the Self (smoking cannabis does not help me to concentrate); Utilitarian Gains for Others (my cannabis use bothers other people) and Self approval (I am embarrassed to smoke cannabis) are specifically endorsed. These dimensions have shown to be associated with positive behavioral changes after treatment interventions in alcohol and tobacco dependent users [12, 17, 18]. Our results suggest that they could be effectively applied during motivational interventions for cannabis dependence.

The Processes of Change findings suggest that severe cannabis users are more likely to use particular behavioral Processes of Change (Self-revaluation and Counter-conditioning), whereas they did not differ from mild cannabis users with regards to cognitive Processes of Change. These behavioral Change Processes have been shown to be associated with reduction of problematic drug use in clinical samples [9– 14]. They are also associated with positive transitions within the stages of change [19, 20]. Therefore, these findings can also inform the design of motivational interventions for cannabis dependence. The implication of these findings is that severe cannabis users may be more sensitive to behavioral versus cognitive ingredients of these motivational interventions. Moreover, they imply that Self-Reevaluation and Counter-conditioning could be immediately applied in treatment-seeking cannabis users at the beginning of the treatment process.

In conclusion, our results are preliminary and need to be complemented by direct research on the treatment pathways of motivational interventions in cannabis dependence. Recent meta-analytical studies have examined these mechanisms, but have focused in the interventions format and design, but not specifically in the motivational ingredients [35, 36]. Our preliminary results can stimulate further research in this topic.

## Supporting information

S1 Table The protocol used in the study. (DOCX)

S1 Data The SPSS data file. (SAV)

## References

[1] European Monitoring Centre for Drugs and Drug Addiction (EMCDDA). The State of the Drug Problem in Europe. Luxembourg, Publications Office of the European Union. Annual Report 2015. Available in http://www.emcdda.europa.eu/countries.

[2] Bruijnzeel AW, Qi X, Guzhva LV, Wall S, Deng JV, Gold MS, et al Behavioral Characterization of the Effects of Cannabis Smoke and Anandamide in Rats. PLoS One 2016; 11(4):e0153327 doi: 10.1371/journal.pone.0153327 27065006 10.1371/journal.pone.0153327 PMC4827836

[3] Enzi B, Lissek S, Edel M-A, Tegenthoff M, Nicolas V, Scherbaum N, et al Alterations of Monetary Reward and Punishment Processing in Chronic Cannabis Users: An fMRI Study. PLoS ONE 2015;10(3): e0119150 doi: 10.1371/journal.pone.0119150 25799565 10.1371/journal.pone.0119150 PMC4370729

[4] González-Pinto A, González-Ortega I, Alberich S, Ruiz de Azúa S, Bernardo M, Bioque M, et al Opposite Cannabis-Cognition Associations in Psychotic Patients Depending on Family History. PLoS ONE 2016; 11(8): e0160949 doi: 10.1371/journal.pone.0160949 27513670 10.1371/journal.pone.0160949 PMC4981356

[5] Van Winkel R, GROUP. Investigators Further Evidence That Cannabis Moderates Familial Correlation of Psychosis-Related Experiences. PLoS ONE 2015;10(9): e0137625 doi: 10.1371/journal.pone.0137625 26384217 10.1371/journal.pone.0137625 PMC4575144

[6] Lorenzetti V, Cousijn J, Solowij N, Garavan H, Suo C, Yücel M, et al The Neurobiology of Cannabis Use Disorders: A Call for Evidence. Front Behav Neurosci 10(86): eCollection 2016 doi: 10.1371/journal.pone.0137625 PMID: 26384217 10.3389/fnbeh.2016.00086 PMC4861711 27242457

[7] Koenders L, Cousijn J, Vingerhoets WAM, van den Brink W, Wiers RW, Meijer CJ, et al Grey Matter Changes Associated with Heavy Cannabis Use: A Longitudinal sMRI Study. PLoS ONE 2016;11(5): e0152482 doi: 10.1371/journal.pone.0152482 27224247 10.1371/journal.pone.0152482 PMC4880314

[8] Troup LJ, Bastidas S, Nguyen MT, Andrzejewski JA, Bowers M, Nomi JS. An Event-Related Potential Study on the Effects of Cannabis on Emotion Processing. PLoS ONE 2016;11(2): e0149764 doi: 10.1371/journal.pone.0149764 26926868 10.1371/journal.pone.0149764 PMC4772908

[9] D'Amico EJ, Houck JM, Hunter SB, Miles JN, Osilla KC, Ewing BA. Group motivational interviewing for adolescents: change talk and alcohol and marijuana outcomes. J Consult Clin Psychol 2015;83(1):68–80. doi: 10.1037/a0038155 25365779 10.1037/a0038155 PMC4324015

[10] Elliott JC, Carey KB, Scott-Sheldon LA. Development of a decisional balance scale for young adult marijuana use. Psychol Addict Behav 2011; 25(1):90–100. doi: 10.1037/a0021743 21261405 10.1037/a0021743 PMC3066293

[11] Prochaska JO, DiClemente CC, Norcross JC. In search of how people change. Applications to addictive behaviors. Am Psychol 1992; 47(9):1102–14. 1329589 10.1037//0003-066x.47.9.1102

[12] Brown JL, DeMartini KS, Sales JM, Swartzendruber AL, DiClemente RJ. Interventions to reduce alcohol use among HIV-infected individuals: a review and critique of the literature. Curr HIV/AIDS Rep 2013;10(4):356–70. doi: 10.1007/s11904-013-0174-8 23990322 10.1007/s11904-013-0174-8 PMC3855569

[13] Redding CA, Prochaska JO, Paiva A, Rossi JS, Velicer W, Blissmer BJ, et al Baseline stage, severity, and effort effects differentiate stable smokers from maintainers and relapsers. Subst Use Misuse 2011; 46(13):1664–74. doi: 10.3109/10826084.2011.565853 21449711 10.3109/10826084.2011.565853 PMC3184208

[14] Sun X, Prochaska JO, Velicer WF, Laforge RG. Transtheoretical principles and processes for quitting smoking: a 24-month comparison of a representative sample of quitters, relapsers, and non-quitters. Addict Behav 2007; 32(12):2707–26. doi: 10.1016/j.addbeh.2007.04.005 17499935 10.1016/j.addbeh.2007.04.005 PMC2080834

[15] Norman GJ, Velicer WF, Fava JL, Prochaska JO. Dynamic typology clustering within the stages of change for smoking cessation. Addict Behav 1998; 23(2):139–53. 9573419 10.1016/s0306-4603(97)00039-7

[16] Prochaska JO, Velicer WF, Rossi JS, Goldstein MG, Marcus BH, Rakowski W, et al Stages of change and decisional balance for 12 problem behaviors. Health Psychol. 1994;13(1):39–46. doi: 10.1037/0278-6133.13.1.39 8168470 10.1037//0278-6133.13.1.39

[17] Cataldo JK, Dubey S, Prochaska JJ. Smoking cessation: an integral part of lung cancer treatment. Oncology 2010;78(5–6):289–301. doi: 10.1159/000319937 20699622 10.1159/000319937 PMC2945268

[18] Velasquez MM, von Sternberg K, Johnson DH, Green C, Carbonari JP, Parsons JT. Reducing sexual risk behaviors and alcohol use among HIV-positive men who have sex with men: a randomized clinical trial. J Consult Clin Psychol 2009; 77(4):657–67. doi: 10.1037/a0015519 19634959 10.1037/a0015519 PMC3737491

[19] Gökbayrak NS, Paiva AL, Blissmer BJ, Prochaska JO. Predictors of relapse among smokers: transtheoretical effort variables, demographics, and smoking severity. Addict Behav 2015; 42:176–9. doi: 10.1016/j.addbeh.2014.11.022 25481450 10.1016/j.addbeh.2014.11.022 PMC4272892

[20] Longmire-Avital B, Golub SA, Parsons JT. Self-reevaluation as a critical component in sustained viral load change for HIV+ adults with alcohol problems. Ann Behav Med 2010; 40(2):176–83. doi: 10.1007/s12160-010-9194-4 20668976 10.1007/s12160-010-9194-4 PMC2939147

[21] Cuenca-Royo AM, Sánchez-Niubó A, Forero CG, Torrens M, Suelves JM, Domingo-Salvany A. Psychometric properties of the CAST and SDS scales in young adult cannabis users. Addict Behav 2012; 37(6):709–15. doi: 10.1016/j.addbeh.2012.02.012 22386300 10.1016/j.addbeh.2012.02.012

[22] Gossop M, Darke S, Griffiths P, Hando J, Powis B, Hall W, et al The Severity of Dependence Scale (SDS): psychometric properties of the SDS in English and Australian samples of heroin, cocaine and amphetamine users. Addiction 1995; 90(5):607–14. doi: 10.1046/j.1360-0443.1995.9056072.x 7795497 10.1046/j.1360-0443.1995.9056072.x

[23] Hides L, Dawe S, Young RM, Kavanagh DJ. The reliability and validity of the Severity of Dependence Scale for detecting cannabis dependence in psychosis. Addiction 2007; 102(1):35–40. doi: 10.1111/j.1360-0443.2006.01669.x 17207121 10.1111/j.1360-0443.2006.01669.x

[24] Martin G, Copeland J, Gates P, Gilmour S. The Severity of Dependence Scale (SDS) in an adolescent population of cannabis users: reliability, validity and diagnostic cut-off. Drug Alcohol Depend 2006; 9(1):90–3. doi: 10.1016/j.drugalcdep.2005.10.014 PMID: 16310973 10.1016/j.drugalcdep.2005.10.014 16310973

[25] Heatherton TF, Kozlowski LT, Frecker RC, Fagerström KO.The Fagerström Test for Nicotine Dependence: a revision of the Fagerström Tolerance Questionnaire. Br J Addict 1991; 86(9):1119–27. 1932883 10.1111/j.1360-0443.1991.tb01879.x

[26] Becoña E, Vázquez FL. The Fagerström Test for Nicotine Dependence in a Spanish sample. Psychol Rep 1998; 83(3 Pt 2):1455–8. doi: 10.2466/pr0.1998.83.3f.1455 10079737 10.2466/pr0.1998.83.3f.1455

[27] Velicer WF, DiClemente CC, Prochaska JO, Brandenburg N. Decisional balance measure for assessing and predicting smoking status. J Pers Soc Psychol. 1985; 48(5):1279–89. doi: 10.1037/0022-3514.48.5.1279 3998990 10.1037//0022-3514.48.5.1279

[28] Girle R, Hitchcock D, McBurney P, Verheij B Decision support for practical reasoning In Reed Chris; Norman Timothy J. Argumentation machines: new frontiers in argument and computation. Argumentation library. 2004, 9 Boston: Kluwer Academic Publishers pp. 55–83. ISBN 1402018118. OCLC 53814623. doi: 10.1007/978-94-017-0431-1_3

[29] Migneault JP, Velicer WF, Prochaska JO, Stevenson JF. Decisional balance for immoderate drinking in college students. Subst Use Misuse 1999; 34(10):1325–46. 10446764 10.3109/10826089909029387

[30] Prochaska JO, Velicer WF, DiClemente CC, Fava J. Measuring processes of change: applications to the cessation of smoking. J Consult Clin Psychol 1998; 56(4):520–8. 3198809 10.1037//0022-006x.56.4.520

[31] Martin RA, Rossi JS, Rosenbloom, D, Monti PM, Rohsenow DJ. Stages and processes of change for quitting cocaine. 1992; Poster presented at the 26th Annual Convention of the Association for the Advancement of Behaviour Therapy. Boston.

[32] Trujols J, Tejero A, Casas M. Estructura factorial, consistencia interna y eficacia discriminativa del inventario de procesos de cambio para adictos a la heroína. Adicciones 1997; 9 (3): 331–45

[33] Tejero A, Trujols J. Evaluación cognitivo conductual y psicodiagnóstica del trastorno por dependencia de opiáceos In Becoña E., Rodríguez A., Salazar I., editors, Drogodependencias. I Introducción. Santiago de Compostela: Servicio de publicaciones e intercambio científico de la Universidad de Santiago de Compostela; 1994 pp. 163–208

[34] Tejero A, Trujols J. Instrumentos clínicos para la evaluación de la dependencia de cocaína Barcelona: Ars Médica; 2003

[35] Cooper K, Chatters R, Kaltenthaler E, Wong R. Psychological and psychosocial interventions for cannabis cessation in adults: a systematic review short report. Health Technol Assess 2015;19(56):1–130. doi: 10.3310/hta19560 26202542 10.3310/hta19560 PMC4781488

[36] Davis ML, Powers MB, Handelsman P, Medina JL, Zvolensky M, Smits JA. Behavioral therapies for treatment-seeking cannabis users: a meta-analysis of randomized controlled trials. Eval Health Prof 2015; 38(1):94–114. doi: 10.1177/0163278714529970 24695072 10.1177/0163278714529970 PMC4429893

