# Supplementary material for: Decisional balance and processes of change in community-recruited with moderate-high versus mild severity of cannabis dependence
Source: PLoS One. 2017 Dec 4;12(12):e0188476. doi: 10.1371/journal.pone.0188476 (PMC5714356; doi:10.1371/journal.pone.0188476)
Supplement: S1 Table — (DOCX) [file pone.0188476.s001.docx]

Supporting information

**S1 Table.**

IDENTIFIER: (last three numbers of the DNI)

SEX:

AGE:

DEGREE:

GROUP:

SDS

This questionnaire allows us to identify and respond to the needs that your cannabis use may be causing and worrying you. Point out the answer that best reflects how you have felt about cannabis use during the past 12 months.

0 1 2 3

1. Did you ever think your use of cannabis was out of control? Never or almost never

Sometime Often Always or nearly always

2. Did the prospect of missing a smoke make you very anxious or worried? Never or almost never

Sometime Often Always or nearly always

3. Did you worry about your use of cannabis? Not at all A little Quite a lot A great deal

4. Did you wish you could stop? Never or almost never

Sometime Often Always or nearly always

5. In particular to the last 12 months, how difficult would you find it to stop or go without? Not difficult Quite difficult Very difficult Impossible

Fagerström´s Test (FTND)

1. How soon after you wake do you smoke your first cigarette?

Within 5 minutes  (3)

6 - 30 minutes  (2)

31 y 60 minutes  (1)

After 60 minutes  (0)

2. Do you find it difficult to refrain from smoking in places where it is forbidden such as church, the library, or movie theatres?

Yes  (1)

No  (0)

3. Which cigarette would you hate most to give up?

The first one in the morning  (1)

All others (0)

4. How many cigarettes do you smoke? (20 cigarettes are in a pack)

10 or less  (0)

11-20  (1)

21-30  (2)

31 or more  (3)

5. Do you smoke more frequently during the first hours after waking than the rest of the day?

Yes  1)

No  (0)

6. Do you smoke if you are so ill that you are in bed most of the day?

Yes  (1)

No  (0)

Decisional Balance Scale

How important to you at the present time is each of the following statements. Please rate your level of importance on the following 5 point scale from not important at all (1) to extremely important (5). Your rating should reflect how important each statement is in your decision whether to drink or use drugs at the present time.

If you have not used any drug except alcohol, fill in the column on the right only. If you have used cannabis, rate the statements for their importance to your drinking in the column on the right and for your drug use in the column on the left. Please answer every question by placing the appropriate number in the box.

1 = Not Important

2 = Slightly Important

3 = Moderately Important

4 = Very Important

5 = Extremely Important

Cannabis 1 2 3 4 5

1. Taking cannabis relaxes me

2. Using drugs is bad for my health

3. I am more pleasant to be around when I’m taking cannabis

4. My cannabis use causes problems with others

5. I like myself better when I am taking

6 I’m foolish to ignore the warnings about the problems caused by cannabis

7. Consumption is a tradition in my family

8. Because I continue to use cannabis, some people think I lack the character to quit

9. Using cannabis helps me deal with problems

10. I often wake up feeling “hungover” or sick

11. If I try to stop using cannabis I’ll probably be irritable and a pain to be around

12. People close to me would suffer if I become ill from using cannabis

13. By continuing to use cannabis I feel I am making my own decisions

14. Having to lie to others about my cannabis use bothers me

15. I would lose my friends if I stopped using cannabis

16. Some people try to avoid me when I use cannabis

17. Using cannabis helps me to have fun and socialize

18. Cannabis use interferes with my functioning at home and/or at work

19. When I use cannabis I get less angry and less frustrated with others

20. I seem to argue and fight more if I’m using cannabis

21. Using cannabis makes me more of a fun person

22. I feel like I’m a slave to cannabis

23. I feel like one of the gang if I use cannabis along with everybody else

24. Some people close to me are disappointed in me because of my cannabis habit

25. Using cannabis helps me to loosen up and express myself

26. I seem to get myself into trouble when using cannabis

27. Things seem to go better at home and at work when I’m using cannabis

28. I could accidentally hurt someone because of my cannabis

29. I feel I am in control of my cannabis habit

30. I’m embarrassed that I use cannabis too much

31. Not using cannabis at a social gathering would make me feel too different

32. I am losing the trust and respect of my co-workers and/or spouse because of my

or drug cannabis

33. My cannabis use helps give me energy and keep going

34. My cannabis use could kill me

35. I am more sure of myself when I am using cannabis

36. I am setting a bad example for others with my cannabis use

37. Without cannabis my life would be boring and dull

38. My cannabis use makes me feel out of control

39. People seem to like me better when I am using cannabis

40. My cannabis use could land me in trouble with law

41. My cannabis use makes other problems seem less important or problematic

42. I cannot imagine someone being happy without the use of cannabis

Processes of Change Questionnaire

Each statement below describes a situation or thought that you might use to help you not use illegal drugs during the past week. instructions: there are five possible responses to each of the items in the questionnaire: 0=never 1=seldom 2=occasionally 3=frequently 4=repeatedly please read each statement and circle the number on the right to indicate how often you make use of a particular situation or thought to help you not use illegal drugs. Remember these statements refer to situations or thoughts you might use during the past week

Cannabis 0 1 2 3 4

1. I feel disgusted when I think about my drug use

2. I think about how my drug use hurts the people around me

3. Keeping me busy reduces my desire to consume

4. I avoid the people with whom I consumed

5. When I want to consume, I try to distract myself by doing something else

6. I have someone who listens to me when I need to talk about my relationship with cannabis use

7. Through the media (T.V., newspapers, radio, etc.) I know the campaigns that are being carried out against the cannabis use

8. I spend time with people who congratulate me or reward me for not consuming

9. My drug use makes me feel disgusted with myself

10. Some people try to make me feel good when I do not use

11. I am fully aware of the damage that my drug use has done to the people that matter to me

12. I see posters in public places encouraging people to go from drug

13. I have heard that cannabis use can cause great changes in mood and depression

14. I think that drug use hurts the people around me

15. When I depend on drugs I feel ashamed and disappointed with myself

16. I usually put around places where I spend more hours (my house, my room, my place of work, etc.) things that remind me that I should not consume

17. I find it useful to do physical activity to support the desire to consume

18. I avoid going to have fun to places where I know people are going to consume

19. I have someone who tries to share with me their personal experiences with consumption

20. There are people in my daily life who worry that I feel good when I do not use

21. I think about information from articles and advertisements on how to stop smoking

22. I promise not to resort to drugs at times when I feel anxious or insecure of myself

23. I feel scared by the intensity of the desire to consume

24. I tell myself can choose to smoke or not.

25. It affects me emotionally (I feel tense, worried, etc.) when they warn me about relationship problems (family, couple or friendship) that involves using cannabis

26. I avoid going to have fun to places where I know people are going to consume

27. I realize that some people who are stopping to use express their desire not to be incited

28. I recall articles dealing with the problem of quitting smoking

29. I tell myself that I do not need to consume to feel good

30. I know someone I can count on when I have problems with cannabis
